# The clinical and economic burden of pneumonia in patients enrolled in Medicare receiving dialysis: a retrospective, observational cohort study

**DOI:** 10.1186/s12882-016-0412-6

**Published:** 2016-12-12

**Authors:** Scott Sibbel, Reiko Sato, Abigail Hunt, Wendy Turenne, Steven M. Brunelli

**Affiliations:** 1 DaVita Clinical Research, 825 South 8th Street, Minneapolis, MN USA; 2 Pfizer Inc, Collegeville, PA USA

**Keywords:** Pneumonia, Infection, Hospitalization, Dialysis, End-stage renal disease, Costs

## Abstract

**Background:**

End-stage renal disease (ESRD) patients receiving dialysis are at particular risk for infection. We assessed the clinical and economic burden of pneumonia in a population of Medicare-enrolled ESRD patients with respect to incidence and case fatality rates, rates of all-cause and cardiovascular hospitalization, and costs.

**Methods:**

Patients received dialysis between 01 January 2009 and 31 December 2011 and were enrolled in Medicare Parts A and B. Pneumonia episodes were identified from institutional and supplier claims. Patients were considered at-risk from first date of Medicare coverage and were censored upon transplant, withdrawal from dialysis, recovery of renal function, loss of Medicare benefits, or death. Linear mixed-effects models were used to assess hospitalization rates and costs over the 3 months prior to and 12 months following pneumonia episodes.

**Results:**

The pneumonia incidence rate for the study period was 21.4 events/100 patient-years; the majority of episodes (90.1%) required inpatient treatment. The 30-day case fatality rate was 10.7%. Compared to month -3 prior to event, rates of all-cause and cardiovascular hospitalization were higher in the month of the pneumonia episode (IRR, 4.61 and 4.30). All-cause admission rates remained elevated through month 12; cardiovascular admission rates remained elevated through month 6. Mean per-patient per-month costs were $10,976 higher in the month of index episode compared to month -3, largely driven by increased inpatient costs, and remained elevated through end of 12-month follow-up.

**Conclusion:**

Pneumonia episodes are frequent among ESRD patients and result in hospitalizations and greater overall costs to Medicare over the following year.

**Electronic supplementary material:**

The online version of this article (doi:10.1186/s12882-016-0412-6) contains supplementary material, which is available to authorized users.

## Background

End-stage renal disease (ESRD) patients receiving dialysis constitute an older population, frequently have multiple comorbid conditions, and their clinical management is complex. ESRD patients are immunocompromised and have a very high frequency of healthcare contact, typically attending in-center hemodialysis treatment 3 times per week in addition to receiving care in a variety of other settings. Consequently, dialysis patients are at elevated risk for both contracting and transmitting infections; infections are the second leading cause of hospitalization and death in ESRD patients, accounting for 0.45 admissions per patient-year and representing one-quarter of all admissions among hemodialysis patients in 2011–2012 [1].

Approximately 20% of infections in ESRD patients are attributable to pulmonary causes, including pneumonia, and evidence suggests that episodes of pneumonia are associated with particularly high morbidity and mortality in this population [2–4]. However, many of the studies assessing pneumonia burden in ESRD patients are based on data from 10 or more years ago, or are focused on specific patient subgroups (eg, incident dialysis patients); thus, contemporary information on the burden of pneumonia in the overall ESRD patient population is limited.

Episodes of pneumonia frequently result in the need for hospitalization and increased healthcare utilization. Under current reimbursement, costs of hospitalizations and other outpatient and ancillary services incurred as a consequence of pneumonia episodes are borne by the payor—Medicare for the vast majority of ESRD patients—and likely represent a significant economic burden in this already costly patient population. Several studies have reported the estimated costs of pneumonia for Medicare beneficiaries in general [5–7] but have not specifically addressed the economic impact in ESRD patients, for whom costs might be expected to be higher.

In this study, we have assessed both the clinical and economic burden of pneumonia in a contemporary population of Medicare beneficiaries with ESRD.

## Methods

This was a retrospective, observational cohort study. Data were taken from the electronic health records of a large dialysis organization (LDO) and from the United States Renal Data System (USRDS) database, which includes Medicare Part A and B claims. Analyses were conducted retrospectively using deidentified patient data; thus, this study was deemed exempt from the requirement of ethical approval by the institutional review board (Quorum Review, Seattle, WA, USA). We adhered to the Declaration of Helsinki; informed consent was not required.

Analyses were conducted in increasingly restrictive cohorts based on availability of necessary data elements (Additional file 1: Figure S1).

### Analyses of pneumonia incidence and episode length

Patients included were those who between 01 January 2009 and 31 December 2011: 1) received dialysis at the LDO; 2) concurrently, were enrolled in Medicare Parts A and B; and 3) were ≥18 years. Patients were considered at-risk from the first date on which enrollment criteria were met until end of study (31 December 2011) or censoring (for transplant, withdrawal from dialysis, recovery of renal function, loss of Medicare benefits, or death).

Pneumonia episodes were identified from Medicare claims considering ICD-9 diagnosis codes 480.x-486.x or 487.0. Pneumonia was ascribed if there was an inpatient claim with pneumonia diagnosis code in any position or an outpatient claim with pneumonia diagnosis in any position and (±14 days) a claim for chest x-ray (Current Procedural Terminology [CPT] claim code 71010-71035) [7]. The date of the corresponding pneumonia claim was considered the episode start date. Thereafter, consecutive pneumonia claims were considered to represent a single pneumonia episode until a 90-day period had elapsed during which there were no additional pneumonia claims. Any subsequent pneumonia claim after this 90-day period was considered to represent a separate pneumonia episode.

Pneumonia episodes were classified based on the treatment setting: episodes that required inpatient treatment were defined as those in which at least one inpatient claim was identified; outpatient episodes were those with no inpatient claim throughout the entire pneumonia episode.

### Analyses of case fatality rates

These analyses considered the subset of the above patients who also: 1) had at least one qualifying pneumonia event during the study period and 2) had received hemodialysis at LDO within the 90-day period preceding the qualifying pneumonia event. The latter criterion was to ensure visibility to death date, which was abstracted from LDO’s electronic health records.

Patients were eligible to contribute multiple qualifying pneumonia events. For each event, the at-risk period began on the episode start date and continued until censoring (criteria above).

### Analyses of hospitalizations and healthcare expenditures

These analyses considered patients who 1) had index pneumonia event on or before 31 December 2010 and 2) had a 90-day pneumonia-free period preceding the episode to ensure a baseline pneumonia-free control period against which post-pneumonia events could be compared. For patients with multiple qualifying pneumonia events, one was selected at random.

Hospitalizations and healthcare expenditures were compared between a control pneumonia-free period (primary analysis: month -3 preceding index pneumonia; sensitivity analysis months -3 to -1 preceding index pneumonia) and the period following index pneumonia. Follow-up continued for 12 months or until censoring.

Hospitalizations were identified from Medicare Part A claims. Cardiovascular hospitalizations were the subset of hospitalizations for which the primary diagnosis was cardiovascular-related (Additional file 1: Table S1).

Healthcare cost data were considered from a Medicare perspective and were derived from claims data and considered as cumulative monthly paid amounts. Inpatient and ancillary service costs were derived from Medicare Part A Institutional Claims Standard Analytic Files (SAFs). Outpatient costs and dialysis costs were derived from both Medicare Part A outpatient files and Part B Physician/Supplier files.

### Statistical analysis

Patient characteristics were described as means, standard deviations (SDs), medians, and interquartile ranges for continuous variables and as frequencies and proportions for categorical variables.

Pneumonia incidence rates were calculated as rates (number of episodes/time at risk) and were estimated with Poisson 95% confidence intervals (CIs). Episode length was calculated as the number of days between the episode start date and episode end date. For outpatient episodes where only a single claim was observed, mean episode length was specified as 1 day. Case fatality rates were calculated as the number of all-cause deaths that occurred within 30 days and within 180 days of the date of pneumonia diagnosis divided by the total number of pneumonia episodes.

For qualifying pneumonia episodes, all-cause and acute cardiovascular event hospital admission rates and healthcare costs were calculated in each month over the 3 months prior to and 12 months following the episode index date.

Hospitalization rates and costs before and after pneumonia episodes were compared using linear mixed-effects models including an indicator for sequential month and a random-effects term for the unique patient identifier. Models of hospitalization rates were specified with a log link and a Poisson/negative binomial distribution; cost models were specified with an identity link and a Gaussian distribution. Costs were empirically transformed to meet distributional assumptions and results were back-transformed and expressed on the native scale. For all models, primary analyses assessed monthly estimates referent to month -3 values. Sensitivity analyses comparing monthly mean estimates to the mean value over the 3-month period prior to diagnosis (month -3 to month -1) were also performed.

All analyses were performed using SAS^®^ 9.3 (SAS Institute Inc, Cary, NC).

## Results

### Pneumonia incidence and episode length

Baseline characteristics of all eligible patients (*N* = 231,202) are presented in Table 1. The mean age was 61.0 years (SD, 15.3), and 56.4% were male. Patients were predominantly white (45.8%) or black (34.4%) and had been on dialysis for 30.1 months on average.

**Table 1:** Baseline Characteristics of Study Cohorts

	All eligible patients^a^ *N* = 231,202	Patients with pneumonia episodes while dialyzing at LDO^b^ *N* = 39,988
Age, years		
mean ± SD	61.0 ± 15.3	64.6 ± 14.9
median (p25, p75)	62 (51, 72)	66 (55, 76)
Sex, *n* (%)		
Male	130,255 (56.4)	21,602 (54.0)
Female	100,738 (43.6)	18,383 (46.0)
Race/Ethnicity, *n* (%)		
White	105,733 (45.8)	18,426 (46.1)
Black	79,503 (34.4)	13,314 (33.3)
Hispanic	29,028 (12.6)	5408 (13.5)
Asian	6634 (2.9)	1065 (2.7)
Other/unknown	10,095 (4.4)	1772 (4.4)
Dialysis vintage, months		
mean ± SD	30.1 ± 39.6	44.1 ± 41.2
median (p25, p75)	18 (1, 44)	34 (14, 62)
Body mass index, kg/m^2^		
mean ± SD	28.1 ± 7.3	26.9 ± 7.0
median (p25, p75)	26.7 (22.9, 31.8)	25.6 (22.1, 30.3)
Body weight, kg		
mean ± SD	81.3 ± 22.9	77.0 ± 21.4
median (p25, p75)	77.6 (65.1, 93.5)	73.5 (62.1, 88.0)
Access type, *n* (%)		
Arteriovenous fistula	53,819 (23.3)	18,953 (47.4)
Arteriovenous graft	21,322 (9.2)	8302 (20.8)
Central venous catheter	37,334 (16.2)	10,588 (26.5)
Peritoneal dialysis catheter	8953 (3.9)	2122 (5.3)
Unknown	109,565 (47.4)	20 (0.1)
Etiology end-stage renal disease, *n* (%)		
Diabetes	97,779 (42.3)	18,769 (46.9)
Hypertension	66,305 (28.7)	11,341 (28.4)
Other/unknown	66,909 (29.0)	9875 (24.7)
Diabetes, *n* (%)	144,827 (62.7)	28,507 (71.3)
Coronary artery disease, *n* (%)	11,503 (5.0)	4118 (10.3)
Atherosclerosis, *n* (%)	7059 (3.1)	2389 (6.0)
Atrial fibrillation, *n* (%)	110 (0.1)	27 (0.1)
Cardiac arrest, *n* (%)	218 (0.1)	88 (0.2)
Ischemic heart disease, *n* (%)	15,340 (6.6)	5266 (13.2)
Cerebrovascular disease, *n* (%)	1067 (0.5)	376 (0.9)
Myocardial infarction, *n* (%)	281 (0.1)	101 (0.3)
Pericarditis, *n* (%)	118 (0.1)	36 (0.1)
Cancer (any cause), *n* (%)	3010 (1.3)	1020 (2.6)
Congestive heart failure, *n* (%)	15,954 (6.9)	5836 (14.6)
CCI score		
mean ± SD	5.2 ± 1.8	5.9 ± 1.9
median (p25, p75)	5 (4, 6)	6 (5, 7)
Hemoglobin, g/dL		
mean ± SD	11.6 ± 1.4	11.0 ± 1.4
median (p25, p75)	11.6 (10.8, 12.5)	11.1 (10.1, 11.9)
Serum albumin, g/dL		
mean ± SD	3.8 ± 0.5	3.6 ± 0.5
median (p25, p75)	3.8 (3.5, 4.1)	3.7 (3.3, 4.0)
Serum phosphate, mg/dL		
mean ± SD	5.2 ± 1.6	5.0 ± 1.7
median (p25, p75)	5.0 (4.1, 5.9)	4.7 (3.9, 5.8)
Serum calcium, mg/dL		
mean ± SD	8.8 ± 0.7	8.7 ± 0.8
median (p25, p75)	8.8 (8.4, 9.2)	8.7 (8.2, 9.1)
Parathyroid hormone, ng/mL		
mean ± SD	380 ± 394	361 ± 389
median (p25, p75)	271 (176, 446)	261 (166, 419)

*Abbreviations*: *CCI* Charlson comorbidity index, *LDO* large dialysis organization, *p25* 25th percentile, *p75* 75th percentile, *SD* standard deviation

^a^ At time of study entry

^b^ At time of first qualifying pneumonia episode

**Fig. 1:**
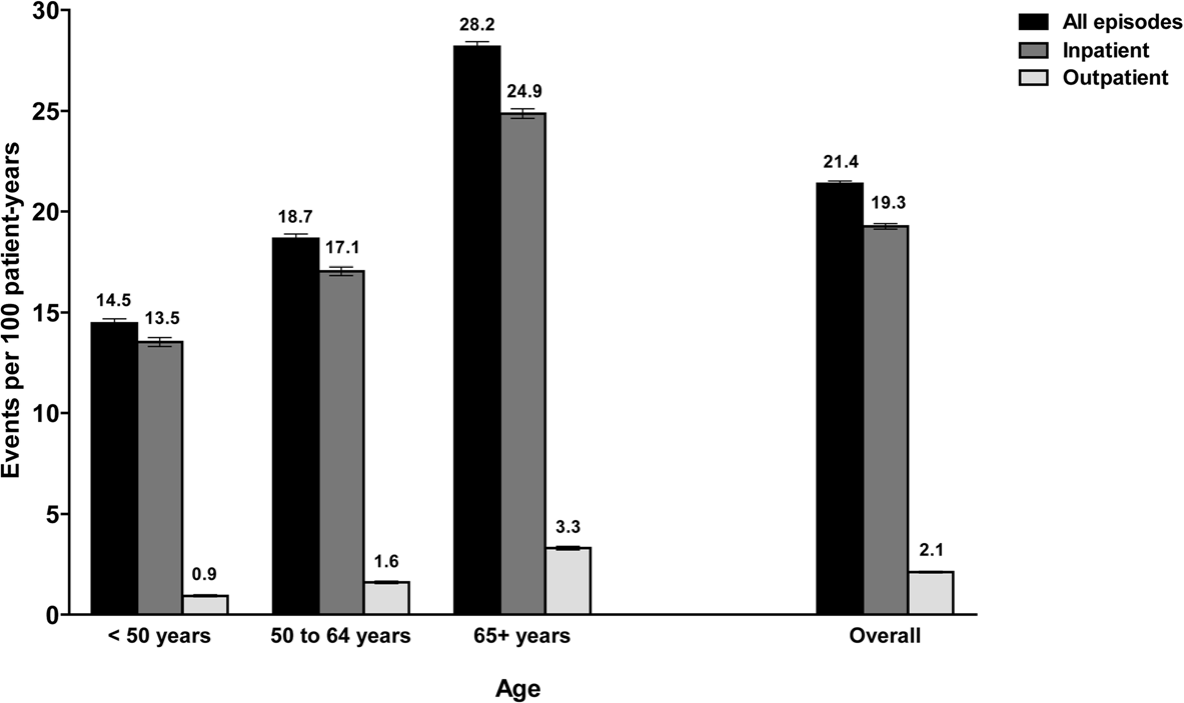
Incidence Rates of Pneumonia (2009–2011)

The overall pneumonia incidence rate was 21.4 events per 100 patient-years (Fig. 1). There were no significant temporal trends in incidence rates across the years of study (2009–2011; data not shown). The majority of episodes (81,883 of 90,862; 90.1%) required inpatient treatment; this corresponded to an incidence rate of 19.3 events per 100 patient-years. A total of 8979 episodes required outpatient treatment only (incidence rate, 2.1 events per 100 patient-years). Stratification by age revealed that incidence rates of pneumonia were higher among older patients.

Overall, the median episode length was 11 days (Table 2). Episodes requiring inpatient treatment were longer (median episode length 12 days), which included a median hospital stay of 8 days. Pneumonia episodes treated solely in the outpatient setting were substantially shorter, with a median length of 1 day, indicating a single pneumonia-related encounter (not including the imaging procedure required definitionally to ascribe pneumonia). Mean episode duration among these patients was 8.3 days, indicating the influence of a small number of protracted episodes.

**Table 2:** Pneumonia Episode Length

	All episodes	Episodes requiring inpatient stay	Episodes treated in outpatient setting only
Number of events	90,862	81,883	8979
Episode length, days			
mean ± SD	29.0 ± 45.9	31.2 ± 47.4	8.3 ± 19.5
median [p25, p75]	11 [5, 33]	12 [6, 36]	1 [1, 8]
Hospitalized days			
mean ± SD		12.8 ± 15.2	
median [p25, p75]		8 [4, 17]	

### Case fatality rates

**Fig. 2:**
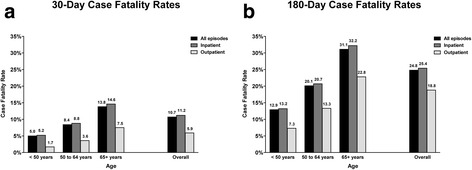
Case Fatality Rates Within 30 Days (panel **a**) and 180 Days (panel **b**) of Pneumonia Episode

The characteristics of 39,988 patients who had pneumonia while concurrently dialyzing at the LDO are presented in Table 1. Among these patients, the overall 30- and 180-day case fatality rates were 10.7% and 24.8%, respectively (Fig. 2a and b). Stratifying by age revealed large differences in case fatality rates across the age groups, with rates increasing as a function of age. Case fatality rates were higher for episodes requiring inpatient treatment compared to episodes treated solely in the outpatient setting (30-day rates of 11.2% vs 5.9% and 180-day rates of 25.4% vs 18.8%, respectively).

### Hospitalizations and healthcare expenditures

**Fig. 3:**
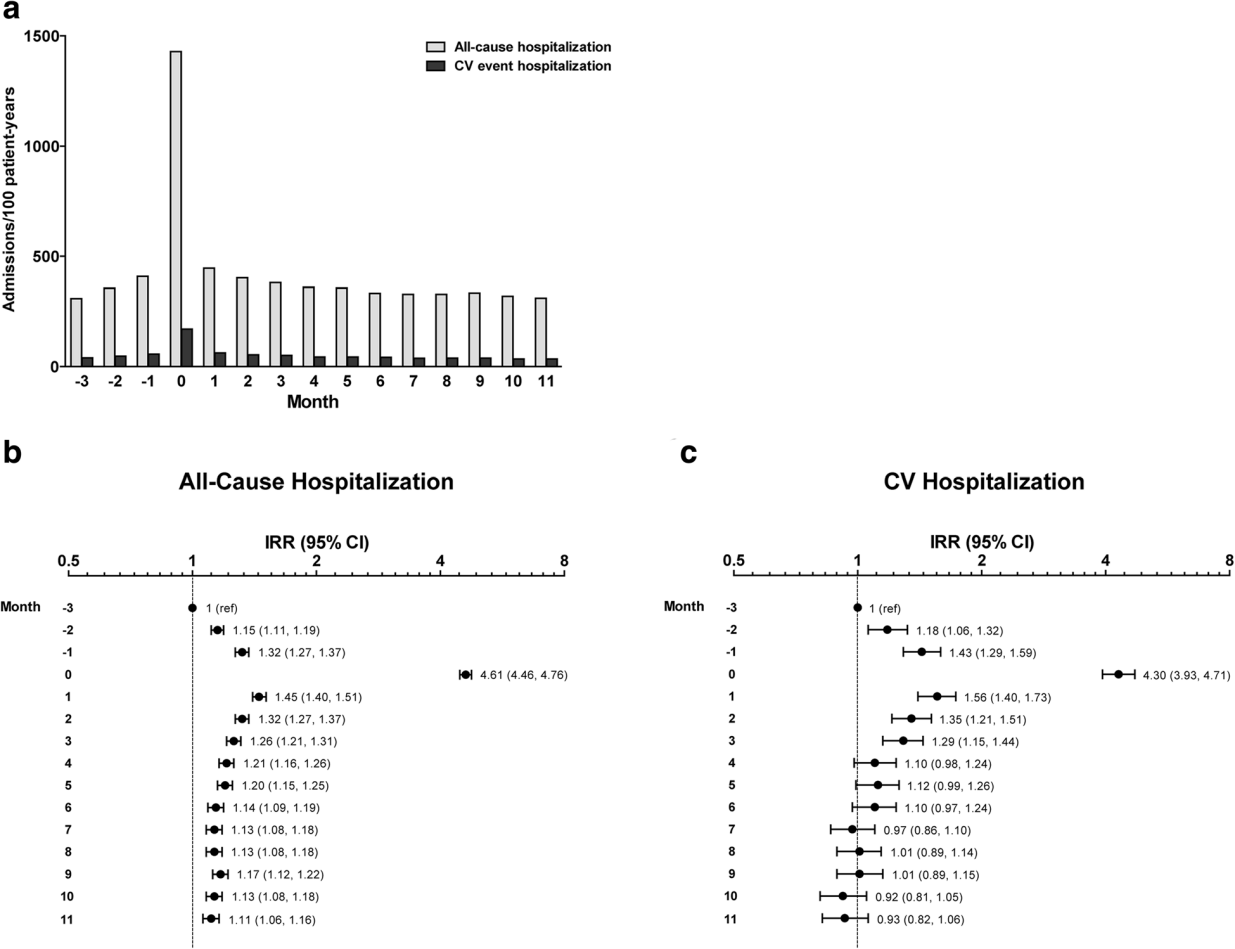
All-Cause and Cardiovascular Event Hospitalization in the Peripneumonia Period. Panel **a** shows absolute rate of hospitalization and CV hospitalization. Panel **b** shows relative rates of all-cause hospitalization relative to month -3. Panel **c** shows relative rates of CV hospitalization relative to month -3. Abbreviations: CI, confidence interval; CV, cardiovascular; IRR, incidence rate ratio; ref, referent

A total of 19,597 patients contributed 1 qualifying pneumonia episode each for comparisons of hospitalizations and healthcare expenditures. Figure 3 and Additional file 1: Table S2 show the rates of all-cause hospital admission and admission for cardiovascular events before and after pneumonia episodes. Crude rates of all-cause hospital admission increased from 308.5 per 100 patient-years in month -3 to 409.1 events per 100 patient-years in month -1 and peaked in the month of index pneumonia episode at 1428.5 events per 100 patient-years (IRR, 4.61; 95% CI, 4.46, 4.76, referent to month -3). Rates declined thereafter, but remained significantly elevated relative to baseline over the 1-year follow-up period. Cardiovascular event hospitalization rates followed a similar pattern, with crude rates increasing from 39.6 to 56.6 admissions per 100 patient-years from month -3 to month -1, reaching 170.5 admissions per 100 patient-years in the month of the pneumonia episode (IRR, 4.30; 95% CI, 3.93, 4.71, referent to month -3). Cardiovascular admission rates declined thereafter and were no longer significantly elevated compared to baseline by month +3 and normalized by month +7.

Sensitivity analyses showed that in the month of index pneumonia event, all-cause hospitalization rates were approximately 4-fold higher (IRR, 3.97; 95% CI, 3.89, 4.04) and cardiovascular event hospitalization rates were greater than 3-fold higher (IRR, 3.55; 95% CI, 3.35, 3.76) than during the 3-month period immediately preceding pneumonia (ie, months -3 to -1; Additional file 1: Table S2).

**Fig. 4:**
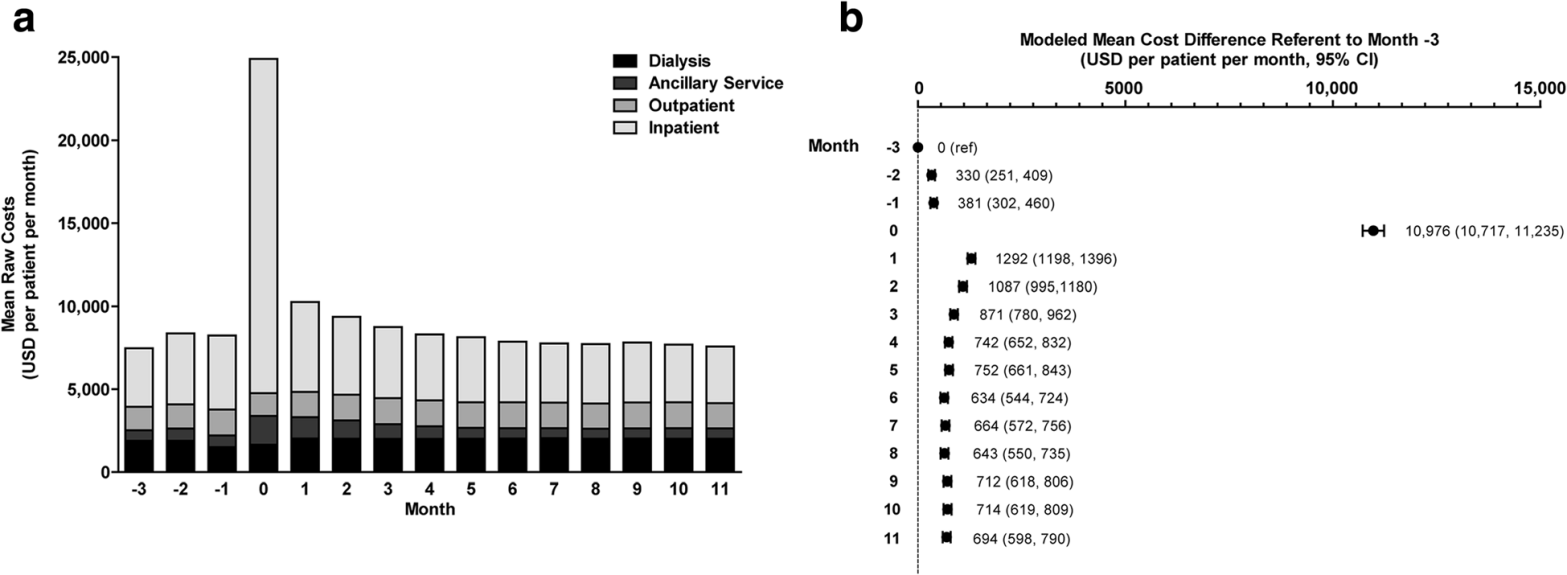
Overall and Component Costs in the Peripneumonia Period. Panel **a** shows mean raw component costs. Panel **b** shows difference in overall monthly costs relative to month -3. Abbreviations: CI, confidence interval; USD, US dollars

Data for component and aggregate healthcare costs are presented in Fig. 4a and Additional file 1: Tables S3 and S4. Monthly inpatient costs increased from a mean of $3522 in month -3 to $20,131 in the month of index pneumonia episode. Costs remained elevated for several months thereafter, gradually declining to pre-pneumonia levels by month 6. With the exception of the month of the index pneumonia episode, median monthly inpatient costs were stable, indicating that mean costs were primarily driven by a small number of expensive events in a minority of patients each month. Outpatient costs remained relatively consistent over time. Mean monthly ancillary service costs increased from $635 in month -3 to $1738 in the month of pneumonia diagnosis and remained elevated relative to pre-episode levels through month +5. Again, median costs were unchanged through the entire period, suggesting that increased mean costs were driven primarily by larger increases for some patients. Dialysis costs fell slightly during the month of the index event, probably due to treatments missed while patients were hospitalized. Thereafter, costs associated with dialysis treatments were elevated over baseline by approximately $100 per month.

Modeled mean incremental costs in each month compared to baseline are shown in Fig. 4b and Additional file 1: Table S4. Referent to month -3, mean total costs were $330 and $381 per patient per month higher in months -2 and -1, and $10,976 higher in the month of index pneumonia episode. Costs declined subsequently but remained $600 to $800 higher relative to month -3 through the end of 12-month follow-up. Sensitivity analyses revealed a mean cost increase of $10,741 per patient per month in the month of the index episode versus the month -3 to -1 mean value. Again, cost differences declined in subsequent months, but remained $400 to $600 greater than the baseline period through the end of the study.

## Discussion

In this study, we have used a merged data set combining USRDS data with LDO electronic health records to enable a comprehensive assessment of the burden of pneumonia in the dialysis population over the period 2009–2011 with respect to incidence rates, case fatality, subsequent hospitalization rates, and costs to Medicare.

The overall incidence rate of pneumonia for the study period was 21.4 events per 100 patient-years and is similar to rates that have been previously published for dialysis patients. For example, Guo et al. reported an incidence rate of 27.9 episodes per 100 patient-years in incident dialysis patients over the period 1996–2001 [2]. The slightly lower rate observed in the current study likely reflects the fact that our study was not limited to incident dialysis patients. Two studies examining community-acquired pneumonia (CAP) in the general Medicare fee-for-service population reported incidence rates of 4.5 and 4.7 per 100 patient-years for the time periods 2007–2008 and 2005–2007, respectively [6, 7]. The dramatically greater pneumonia incidence rate in dialysis patients is probably the result of multiple, interacting factors. Patients with ESRD have a greater comorbidity burden than the general population. Dialysis patients are also at particularly high risk for disease transmission due to frequent healthcare contact. Finally, these patients have compromised immune function, which makes them more susceptible to infections [8]. In comparing the estimates of pneumonia incidence rates reported here to those reported elsewhere in the literature, it also should be noted that our study was not restricted to CAP episodes; given that dialysis patients spend up to 12 h per week in healthcare facilities, the distinction between community-acquired and nosocomial disease may be artificial in this patient population.

The proportion of pneumonia episodes requiring inpatient treatment in our study was approximately 90%, which was noticeably higher than other studies. Previous studies have reported that inpatient cases comprised 42% of pneumonia episodes for dialysis patients [2] and 39 to 47% for the general Medicare population [6, 7]. The lower rate of episodes requiring inpatient treatment in the general Medicare population is not surprising, given the greater comorbidity burden of the ESRD population. It is likely that the difference between our estimate and those from prior studies of ESRD patients is, at least in part, due to differences in operational definitions of pneumonia episodes.

We observed 30- and 180-day case fatality rates for pneumonia episodes overall of 10.7 and 24.8%, respectively. Rates increased with patient age and were higher for episodes that required inpatient care than for those treated solely in the outpatient setting. These rates are generally consistent with several prior studies of ESRD patients [2–4].

Among patients with a qualifying pneumonia episode, we observed rates of all-cause hospitalization that were 4.6-fold higher in the month of the index pneumonia episode compared to the month -3 baseline. Rates of hospitalization for acute cardiovascular events were also observed to increase over 4-fold in the month of pneumonia diagnosis. These substantial increases are particularly striking, considering that the cohort of dialysis patients who developed pneumonia already had elevated baseline hospitalization rates—at 309 admissions/100 patient-years in month -3, compared to 174 admissions per 100 patient-years reported for the hemodialysis population in 2012 [1]. The fact that patients who developed pneumonia were generally older, and had lower body mass index, serum albumin, and hemoglobin levels than the study cohort overall provides a plausible explanation for these elevated baseline admission rates. All of these characteristics are typical of the protein-energy wasting/malnutrition-inflammation complex, which is common among patients with chronic kidney disease and has been shown to be associated with increased overall and cardiovascular morbidity and mortality risk [9].

The observed increase in cardiovascular hospitalization rate following pneumonia episodes confirms that not all of the increase in hospital admissions can be attributed to pneumonia-related events and, moreover, is consistent with accumulating evidence of a relationship between pneumonia and increased risk of subsequent cardiovascular complications [2, 10, 11]. While the exact mechanism linking pneumonia with cardiovascular events has yet to be determined, the persistent inflammatory state induced by infections such as pneumonia and sepsis is a leading hypothesis, and infections have specifically been linked to cardiovascular mortality risk in dialysis patients [12–14].

Prior studies of the economic consequences of pneumonia have focused on the general Medicare population and have not specifically addressed costs in ESRD patients [5–7]. The total incremental cost, which considered all inpatient, outpatient, ancillary, and dialysis services costs relative to utilization in month -3 prior to pneumonia diagnosis, accumulated to approximately $20,000 over the year following a pneumonia episode. Month -3 was selected as the referent month for primary analyses to account for increases in healthcare utilization that may occur in the months immediately prior to pneumonia diagnosis. However, when costs were considered relative to the mean of the 3-month period comprising months -3 to -1— ie, a more conservative approach—the incremental costs still amounted to approximately $17,000. Incremental costs were largely driven by high inpatient costs incurred within the 3 months following the pneumonia diagnosis. However, there are clearly longer-term economic consequences as well, given that total costs remained elevated over baseline through the end of the 12-month follow-up period.

Our study was not without limitations. Administrative claims data were used for these analyses and may not be complete in all cases. Pneumonia episodes were defined considering consecutive claims for pneumonia as representing a single episode until a period of 90 days had elapsed, during which there were no claims for pneumonia; any comparison of the incidence rates presented here with rates derived from other studies should take into account differences in episode definitions. To assess hospitalizations and healthcare costs following pneumonia episodes, a 90-day pneumonia-free baseline period was required for episodes to be included. Although this limited our analysis to patients with episodes meeting these criteria, the study design meant that patients served as their own controls, eliminating the need for covariate adjustment. Our analyses of the economic consequences of pneumonia were considered in the context of current reimbursement and pertain only to Medicare patients in the US. Finally, we were not able to identify the etiologic organism responsible for pneumonia episodes, as this information was not present in the administrative data used for this study.

## Conclusions

Overall, the results of our study demonstrate that pneumonia poses a significant clinical and economic burden among ESRD patients. More than 1 in 5 patients die within 180 days of pneumonia diagnosis, and hospitalization rates remain elevated over baseline rates for at least 1 year. Based on the incidence rates and incremental costs observed here, the overall annual cost to Medicare associated with pneumonia among ESRD patients is very substantial. Thus, increased focus on all types of pneumonia prevention measures—including vaccination—particularly among the frailest patients, clearly represents an opportunity to improve outcomes among ESRD patients receiving dialysis.

## Additional file

Additional file 1: Figure S1. Study Cohorts. **Table S1.** ICD-9 Codes for Cardiovascular Event. **Table S2.** All-Cause and Cardiovascular Event Hospitalization Rates Before and After Pneumonia Episodes. **Table S3.** Healthcare Costs Before and After Pneumonia Episodes. **Table S4.** Modeled Differences in Total Costs Versus Baseline. (DOCX 76 kb)

## Abbreviations

CAP: Community- acquired pneumonia
CI: Confidence interval
CPT: Current procedural terminology
ESRD: End- stage renal disease
IRR: Incidence rate ratio
LDO: Large dialysis organization
SAF: Standard analytic files
SD: Standard deviation
USRDS: United States Renal Data System
