# Supplementary material for: The clinical and economic burden of pneumonia in patients enrolled in Medicare receiving dialysis: a retrospective, observational cohort study
Source: BMC Nephrol. 2016 Dec 12;17:199. doi: 10.1186/s12882-016-0412-6 (PMC5153919; doi:10.1186/s12882-016-0412-6)
Supplement: Additional file 1: Figure S1. — Study Cohorts. Table S1. ICD-9 Codes for Cardiovascular Event. Table S2. All-Cause and Cardiovascular Event Hospitalization Rates Before and After Pneumonia Episodes. Table S3. Healthcare Costs Before and After Pneumonia Episodes. Table S4. Modeled Differences in Total Costs Versus Baseline. (DOCX 76 kb) [file 12882_2016_412_MOESM1_ESM.docx]

**Additional file**

**Additional file 1: Figure S1: Study Cohorts**

**
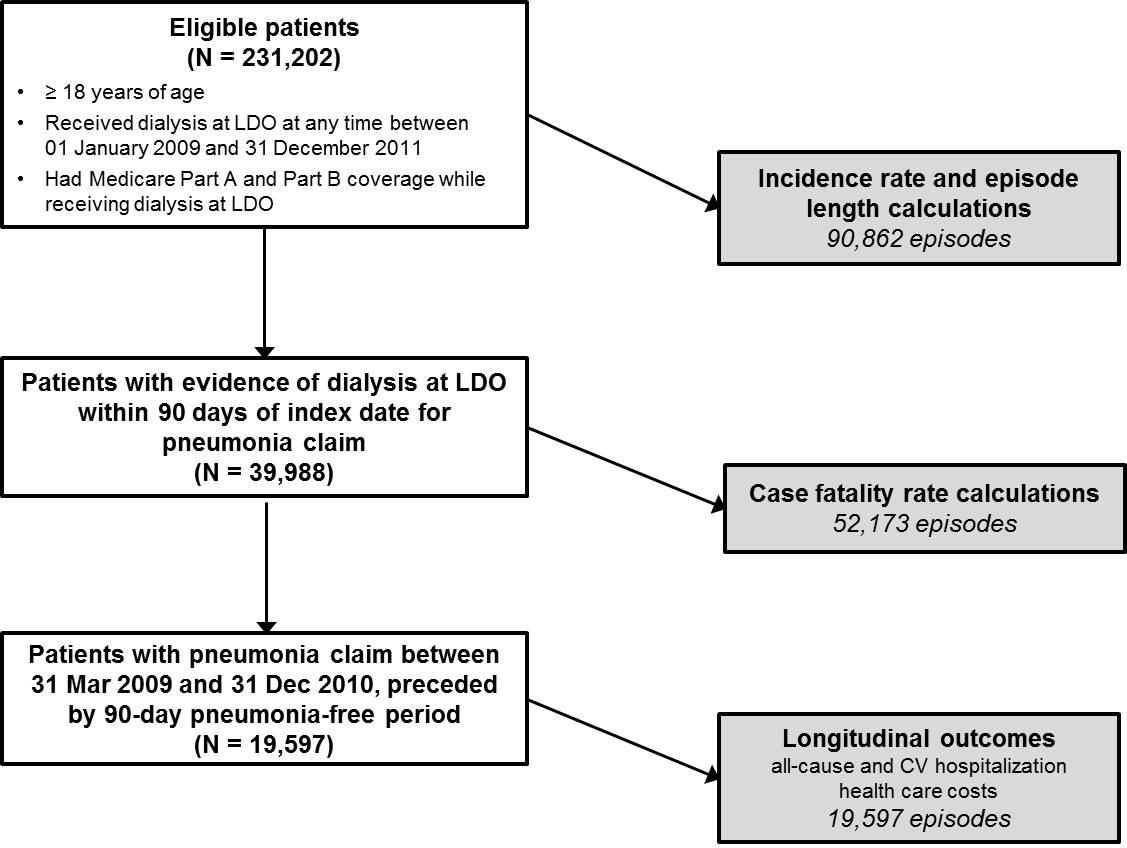
**

**Additional file 1: Table S1: ICD-9 Codes for Cardiovascular Event**

| **ICD-9 Code** | **Long Description** |
| --- | --- |
| \| 410 \| \| --- \| \| 4100 \| \| 41000 \| \| 41001 \| \| 41002 \| \| 4101 \| \| 41010 \| \| 41011 \| \| 41012 \| \| 4102 \| \| 41020 \| \| 41021 \| \| 41022 \| \| 4103 \| \| 41030 \| \| 41031 \| \| 41032 \| \| 4104 \| \| 41040 \| \| 41041 \| \| 41042 \| \| 4105 \| \| 41050 \| \| 41051 \| \| 41052 \| \| 4106 \| \| 41060 \| \| 41061 \| \| 41062 \| \| 4107 \| \| 41070 \| \| 41071 \| \| 41072 \| \| 4108 \| \| 41080 \| \| 41081 \| \| 41082 \| \| 4109 \| \| 41090 \| \| 41091 \| \| 41092 \| \| 411 \| \| 4110 \| \| 4111 \| \| 4118 \| \| 41181 \| \| 41189 \| \| 412 \| \| 413 \| \| 4130 \| \| 4131 \| \| 4139 \| \| 414 \| \| 4140 \| \| 41400 \| \| 41401 \| \| 41403 \| \| 41406 \| \| 4142 \| \| 4143 \| \| 4148 \| \| 4149 \| \| 426 \| \| 4260 \| \| 4261 \| \| 42610 \| \| 42611 \| \| 42612 \| \| 42613 \| \| 4262 \| \| 4263 \| \| 4264 \| \| 4265 \| \| 42650 \| \| 42651 \| \| 42652 \| \| 42653 \| \| 42654 \| \| 4266 \| \| 4267 \| \| 4268 \| \| 42681 \| \| 42682 \| \| 42689 \| \| 4269 \| \| 427 \| \| 4270 \| \| 4271 \| \| 4272 \| \| 4273 \| \| 42731 \| \| 42732 \| \| 4276 \| \| 42760 \| \| 42761 \| \| 42769 \| \| 4278 \| \| 42781 \| \| 42789 \| \| 4279 \| \| 7850 \| \| 7851 \| \| 443 \| \| 4430 \| \| 4274 \| \| 42741 \| \| 42742 \| \| 4275 \| \| 433 \| \| 4330 \| \| 43300 \| \| 4331 \| \| 43310 \| \| 4332 \| \| 43320 \| \| 4333 \| \| 43330 \| \| 4338 \| \| 43380 \| \| 4339 \| \| 43390 \| \| 437 \| \| 4370 \| \| 4371 \| \| 4373 \| \| 4374 \| \| 4375 \| \| 4376 \| \| 4377 \| \| 4378 \| \| 4379 \| \| 440 \| \| 4400 \| \| 4401 \| \| 4402 \| \| 44020 \| \| 44021 \| \| 44022 \| \| 44023 \| \| 44029 \| \| 41402 \| \| 41404 \| \| 41405 \| \| 41407 \| \| 44024 \| \| 4255 \| \| 425 \| \| 4250 \| \| 4251 \| \| 4252 \| \| 4253 \| \| 4254 \| \| 4257 \| \| 4258 \| \| 4259 \| | \| Acute myocardial infarction \| \| --- \| \| Acute myocardial infarction of anterolateral wall \| \| Acute myocardial infarction of anterolateral wall, episode of care unspecified \| \| Acute myocardial infarction of anterolateral wall, initial episode of care \| \| Acute myocardial infarction of anterolateral wall, subsequent episode of care \| \| Acute myocardial infarction of other anterior wall \| \| Acute myocardial infarction of other anterior wall, episode of care unspecified \| \| Acute myocardial infarction of other anterior wall, initial episode of care \| \| Acute myocardial infarction of other anterior wall, subsequent episode of care \| \| Acute myocardial infarction of inferolateral wall \| \| Acute myocardial infarction of inferolateral wall, episode of care unspecified \| \| Acute myocardial infarction of inferolateral wall, initial episode of care \| \| Acute myocardial infarction of inferolateral wall, subsequent episode of care \| \| Acute myocardial infarction of inferoposterior wall \| \| Acute myocardial infarction of inferoposterior wall, episode of care unspecified \| \| Acute myocardial infarction of inferoposterior wall, initial episode of care \| \| Acute myocardial infarction of inferoposterior wall, subsequent episode of care \| \| Acute myocardial infarction of other inferior wall \| \| Acute myocardial infarction of other inferior wall, episode of care unspecified \| \| Acute myocardial infarction of other inferior wall, initial episode of care \| \| Acute myocardial infarction of other inferior wall, subsequent episode of care \| \| Acute myocardial infarction of other lateral wall \| \| Acute myocardial infarction of other lateral wall, episode of care unspecified \| \| Acute myocardial infarction of other lateral wall, initial episode of care \| \| Acute myocardial infarction of other lateral wall, subsequent episode of care \| \| True posterior wall infarction \| \| True posterior wall infarction, episode of care unspecified \| \| True posterior wall infarction, initial episode of care \| \| True posterior wall infarction, subsequent episode of care \| \| Subendocardial infarction \| \| Subendocardial infarction, episode of care unspecified \| \| Subendocardial infarction, initial episode of care \| \| Subendocardial infarction, subsequent episode of care \| \| Acute myocardial infarction of other specified sites \| \| Acute myocardial infarction of other specified sites, episode of care unspecified \| \| Acute myocardial infarction of other specified sites, initial episode of care \| \| Acute myocardial infarction of other specified sites, subsequent episode of care \| \| Acute myocardial infarction of unspecified site \| \| Acute myocardial infarction of unspecified site, episode of care unspecified \| \| Acute myocardial infarction of unspecified site, initial episode of care \| \| Acute myocardial infarction of unspecified site, subsequent episode of care \| \| Other acute and subacute forms of ischemic heart disease \| \| Postmyocardial infarction syndrome \| \| Intermediate coronary syndrome \| \| Other acute and subacute forms of ischemic heart disease \| \| Acute coronary occlusion without myocardial infarction \| \| Other acute and subacute forms of ischemic heart disease, other \| \| Old myocardial infarction \| \| Angina pectoris \| \| Angina decubitus \| \| Prinzmetal angina \| \| Other and unspecified angina pectoris \| \| Other forms of chronic ischemic heart disease \| \| Coronary atherosclerosis \| \| Coronary atherosclerosis of unspecified type of vessel, native or graft \| \| Coronary atherosclerosis of native coronary artery \| \| Coronary atherosclerosis of nonautologous biological bypass graft \| \| Coronary atherosclerosis of native coronary artery of transplanted heart \| \| Chronic total occlusion of coronary artery \| \| Coronary atherosclerosis due to lipid rich plaque \| \| Other specified forms of chronic ischemic heart disease \| \| Chronic ischemic heart disease unspecified \| \| Conduction disorders \| \| Atrioventricular block, complete \| \| Atrioventricular block, other and unspecified \| \| Atrioventricular block, unspecified \| \| First degree atrioventricular block \| \| Mobitz (type) ii atrioventricular block \| \| Other second degree atrioventricular block \| \| Left bundle branch hemiblock \| \| Other left bundle branch block \| \| Right bundle branch block \| \| Bundle branch block, other and unspecified \| \| Bundle branch block, unspecified \| \| Right bundle branch block and left posterior fascicular block \| \| Right bundle branch block and left anterior fascicular block \| \| Other bilateral bundle branch block \| \| Trifascicular block \| \| Other heart block \| \| Anomalous atrioventricular excitation \| \| Other specified conduction disorders \| \| Lown-Ganong-Levine syndrome \| \| Long qt syndrome \| \| Other specified conduction disorders \| \| Conduction disorder unspecified \| \| Cardiac dysrhythmias \| \| Paroxysmal supraventricular tachycardia \| \| Paroxysmal ventricular tachycardia \| \| Paroxysmal tachycardia, unspecified \| \| Atrial fibrillation and flutter \| \| Atrial fibrillation \| \| Atrial flutter \| \| Premature beats \| \| Premature beats, unspecified \| \| Supraventricular premature beats \| \| Other premature beats \| \| Other specified cardiac dysrhythmias \| \| Sinoatrial node dysfunction \| \| Other specified cardiac dysrhythmias \| \| Cardiac dysrhythmia, unspecified \| \| Tachycardia unspecified \| \| Palpitations \| \| Other peripheral vascular disease \| \| Raynaud’s syndrome \| \| Ventricular fibrillation and flutter \| \| Ventricular fibrillation \| \| Ventricular flutter \| \| Cardiac arrest \| \| Occlusion and stenosis of precerebral arteries \| \| Occlusion and stenosis of basilar artery \| \| Occlusion and stenosis of basilar artery without cerebral infarction \| \| Occlusion and stenosis of carotid artery \| \| Occlusion and stenosis of carotid artery without cerebral infarction \| \| Occlusion and stenosis of vertebral artery \| \| Occlusion and stenosis of vertebral artery without cerebral infarction \| \| Occlusion and stenosis of multiple and bilateral precerebral arteries \| \| Occlusion and stenosis of multiple and bilateral precerebral arteries without cerebral infarction \| \| Occlusion and stenosis of other specified precerebral artery \| \| Occlusion and stenosis of other specified precerebral artery without cerebral infarction \| \| Occlusion and stenosis of unspecified precerebral artery \| \| Occlusion and stenosis of unspecified precerebral artery without cerebral infarction \| \| Other and ill-defined cerebrovascular disease \| \| Cerebral atherosclerosis \| \| Other generalized ischemic cerebrovascular disease \| \| Cerebral aneurysm, nonruptured \| \| Cerebral arteritis \| \| Moyamoya disease \| \| Nonpyogenic thrombosis of intracranial venous sinus \| \| Transient global amnesia \| \| Other ill-defined cerebrovascular disease \| \| Unspecified cerebrovascular disease \| \| Atherosclerosis \| \| Atherosclerosis of aorta \| \| Atherosclerosis of renal artery \| \| Atherosclerosis of native arteries of the extremities \| \| Atherosclerosis of native arteries of the extremities, unspecified \| \| Atherosclerosis of native arteries of the extremities with intermittent claudication \| \| Atherosclerosis of native arteries of the extremities with rest pain \| \| Atherosclerosis of native arteries of the extremities with ulceration \| \| Other atherosclerosis of native arteries of the extremities \| \| Coronary atherosclerosis of autologous vein bypass graft \| \| Coronary atherosclerosis of artery bypass graft \| \| Coronary atherosclerosis of unspecified bypass graft \| \| Coronary atherosclerosis of bypass graft (artery) (vein) of transplanted heart \| \| Atherosclerosis of native arteries of the extremities with gangrene \| \| Alcoholic cardiomyopathy \| \| Cardiomyopathy \| \| Endomyocardial fibrosis \| \| Hypertrophic obstructive cardiomyopathy \| \| Obscure cardiomyopathy of Africa \| \| Endocardial fibroelastosis \| \| Other primary cardiomyopathies \| \| Nutritional and metabolic cardiomyopathy \| \| Cardiomyopathy in other diseases classified elsewhere \| \| Secondary cardiomyopathy, unspecified \| |

Additional file 1: Table S2: All-Cause and Cardiovascular Event Hospitalization Rates Before and After Pneumonia Episodes

|  | **Month** (relative to index pneumonia diagnosis) | | | | | | | | | | | | | | |
| --- | --- | --- | --- | --- | --- | --- | --- | --- | --- | --- | --- | --- | --- | --- | --- |
|  | **-3** | **-2** | **-1** | **0** | **+1** | **+2** | **+3** | **+4** | **+5** | **+6** | **+7** | **+8** | **+9** | **+ 10** | **+11** |
| **All-Cause Hospitalization** | | | | | | | | | | | | | | | |
| No. Events  Pt time  (100 pt-yrs)  Raw rate  (events/100 pt-yrs) | 4728  15.33  308.51 | 5509  15.52  355.01 | 6457  15.78  409.06 | 23,936  16.76  1428.54 | 6579  14.75  445.96 | 5634  13.98  403.05 | 5088  13.37  380.64 | 4627  12.86  359.87 | 4437  12.46  356.23 | 3993  12.03  331.89 | 3830  11.69  327.58 | 3725  11.37  327.56 | 3693  11.07  333.49 | 3449  10.82  318.79 | 3262  10.51  310.38 |
| ***Modeled differences in all-cause hospitalization rates vs baseline*** | | | | | | | | | | | | | | | |
| IRR  (95% CI) | Ref | 1.15  1.11, 1.19 | 1.32  1.27, 1.37 | 4.61  4.46, 4.76 | 1.45  1.40, 1.51 | 1.32  1.27, 1.37 | 1.26  1.21, 1.31 | 1.21  1.16, 1.26 | 1.20  1.15, 1.25 | 1.14  1.09, 1.19 | 1.13  1.08, 1.18 | 1.13  1.08, 1.18 | 1.17  1.12, 1.22 | 1.13  1.08, 1.18 | 1.11  1.06, 1.16 |
| IRR  (95% CI) | Ref | | | 3.97  3.89, 4.04 | 1.25  1.21, 1.28 | 1.14  1.10, 1.17 | 1.09  1.05, 1.12 | 1.04  1.00, 1.07 | 1.04  1.00, 1.07 | 0.98  0.95, 1.02 | 0.97  0.94, 1.01 | 0.98  0.94, 1.01 | 1.00  0.97, 1.04 | 0.98  0.94, 1.01 | 0.96  0.93, 1.00 |
| **Cardiovascular Event Hospitalization** | | | | | | | | | | | | | | | |
| No. Events  Pt time  (100 pt-yrs)  Raw rate  (events/100 pt-yrs) | 607  15.33  39.61 | 730  15.52  47.04 | 894  15.78  56.64 | 2856  16.76  170.45 | 913  14.75  61.89 | 747  13.98  53.44 | 679  13.37  50.80 | 556  12.86  43.24 | 541  12.46  43.44 | 509  12.03  42.31 | 440  11.69  37.63 | 439  11.37  38.60 | 425  11.07  38.38 | 377  10.82  34.85 | 366  10.51  34.82 |
| ***Modeled differences in cardiovascular event hospitalization rates vs baseline*** | | | | | | | | | | | | | | | |
| IRR  (95% CI) | Ref  ^a^ | 1.18  1.06, 1.32 | 1.43  1.29, 1.59 | 4.30  3.93, 4.71 | 1.56  1.40, 1.73 | 1.35  1.21, 1.51 | 1.29  1.15, 1.44 | 1.10  0.98, 1.24 | 1.12  0.99, 1.26 | 1.10  0.97, 1.24 | 0.97  0.86, 1.10 | 1.01  0.89, 1.14 | 1.01  0.89, 1.15 | 0.92  0.81, 1.05 | 0.93  0.82, 1.06 |
| IRR  (95% CI) | Ref  ^a^ | | | 3.55  3.35, 3.76 | 1.28  1.18, 1.39 | 1.11  1.02, 1.21 | 1.06  0.97, 1.16 | 0.91  0.83, 1.00 | 0.92  0.84, 1.02 | 0.91  0.82, 1.01 | 0.81  0.73, 0.90 | 0.84  0.75, 0.93 | 0.84  0.75, 0.93 | 0.77  0.69, 0.85 | 0.77  0.69, 0.86 |
| ^a^ Two referent group approaches were employed: referent month = month -3 and referent month = month -3 to month -1 mean.  Abbreviations: CI, confidence interval; IRR, incidence rate ratio; pt, patient; Ref, referent; yrs, years. | | | | | | | | | | | | | | | |

Additional file 1: Table S3: Health Care Costs Before and After Pneumonia Episodes

| **Costs**  USD PPPM | **Month** (relative to index pneumonia diagnosis) | | | | | | | | | | | | | | | |
| --- | --- | --- | --- | --- | --- | --- | --- | --- | --- | --- | --- | --- | --- | --- | --- | --- |
|  | **-3** | **-2** | **-1** | **0** | | **+1** | **+2** | **+3** | **+4** | **+5** | **+6** | **+7** | **+8** | **+9** | **+ 10** | **+11** |
| **Inpatient Costs** | | | | | | | | | | | | | | | | |
| mean  SD  median  p25, p75 | 3522  11,875  0  0, 0 | 4278  12,545  0  0, 0 | 4468  10,912  0  0, 0 | 20,131  30,140  11,225  7162, 23,626 | 5426  13,897  0  0, 5964 | | 4693  12,772  0  0, 2654 | 4293  11,728  0  0, 0 | 3989  11,501  0  0, 0 | 3929  11,007  0  0, 0 | 3660  11,223  0  0, 0 | 3572  10,643  0  0, 0 | 3580  10,720  0  0, 0 | 3617  10,803  0  0, 0 | 3476  10,133  0  0, 0 | 3423  10,242  0  0, 0 |
| **Outpatient Costs** | | | | | | | | | | | | | | | | |
| mean  SD  median  p25, p75 | 1420  2054  653  362, 1510 | 1459  2123  682  369, 1571 | 1564  2163  769  396, 1760 | 1370  1923  770  389, 1526 | 1522  2154  738  382, 1703 | | 1552  2156  715  383, 1738 | 1578  2253  729  394, 1765 | 1561  2239  707  389, 1706 | 1540  2194  705  384, 1667 | 1565  2280  699  391, 1718 | 1542  2185  685  389, 1659 | 1529  2191  681  388, 1601 | 1562  2191  709  395, 1694 | 1559  2259  694  394, 1649 | 1524  2219  695  397, 1587 |
| **Ancillary Service Costs** | | | | | | | | | | | | | | | | |
| mean  SD  median  p25, p75 | 635  2312  0  0, 0 | 735  2440  0  0, 0 | 711  2152  0  0, 0 | 1738  3838  0  0, 1725 | 1294  3234  0  0, 0 | | 1110  4286  0  0, 0 | 880  2572  0  0, 0 | 756  2270  0  0, 0 | 660  2153  0  0, 0 | 625  2059  0  0, 0 | 602  2076  0  0, 0 | 598  2038  0  0, 0 | 615  2118  0  0, 0 | 637  2190  0  0, 0 | 617  2150  0  0, 0 |
| **Dialysis Costs** | | | | | | | | | | | | | | | | |
| mean  SD  median  p25, p75 | 1892  1500  2053  0, 2709 | 1895  1479  2040  0, 2736 | 1503  1288  1572  0, 2281 | 1654  1599  1754  0, 2671 | 2027  1590  2137  277, 2939 | | 2011  1528  2103  731, 2852 | 2006  1470  2083  981, 2788 | 2006  1449  2075  1177, 2744 | 2016  1406  2099  1256, 2743 | 2021  1472  2089  1304, 2732 | 2045  1428  2102  1408, 2726 | 2017  1338  2085  1429, 2705 | 2022  1375  2091  1437, 2685 | 2018  1354  2091  1423, 2686 | 2019  1321  2099  1469, 2680 |
| **Total Costs to Payor** | | | | | | | | | | | | | | | | |
| mean  SD  median  p25, p75 | 7470  12,519  3510  2278, 8040 | 8367  13,186  3732  2354, 9437 | 8245  11,536  3703  2105, 10,481 | 24,893  29,875  16,979  10,765, 30,082 | 10,269  14,497  4972  2701, 13,106 | | 9367  13,811  4563  2598, 11,805 | 8757  12,466  4171  2521, 10,827 | 8313  12,184  3951  2484, 10,115 | 8145  11,651  3894  2510, 9618 | 7871  11,918  3823  2472, 8996 | 7761  11,297  3694  2474, 8901 | 7725  11,408  3675  2461, 8842 | 7817  11,562  3677  2490, 8818 | 7690  10,920  3641  2469, 8774 | 7583  10,982  3588  2459, 8510 |
| Abbreviations: p25, 25th percentile; p75, 75th percentile; PPPM, per patient per month; SD, standard deviation; USD, US dollars. | | | | | | | | | | | | | | | | |

Additional file 1: Table S4: Modeled Differences in Total Costs Versus Baseline

| **Costs**  USD PPPM | **Month** (relative to index pneumonia diagnosis) | | | | | | | | | | | | | | |
| --- | --- | --- | --- | --- | --- | --- | --- | --- | --- | --- | --- | --- | --- | --- | --- |
|  | **-3** | **-2** | **-1** | **0** | **+1** | **+2** | **+3** | **+4** | **+5** | **+6** | **+7** | **+8** | **+9** | **+ 10** | **+11** |
| Diff ^b^  (95% CI) | Ref ^a^ | +330  +251, +409 | +381  +302, +460 | +10,976  +10,717, +11,235 | +1292  +1198, +1386 | +1087  +995, +1180 | +871  +780, +962 | +742  +652, +832 | +752  +661, +843 | +634  +544, +724 | +664  +572, +756 | +643  +550, +735 | +712  +618, +806 | +714  +619, +809 | +694  +598, +790 |
| Diff ^b^  (95% CI) | Ref ^a^ | | | +10,741  +10,486, +10,995 | +1057  +973, +1141 | +852  +770, +935 | +636  +556, +717 | +508  +428, +587 | +517  +436, +598 | +399  +319, +479 | +429  +348, +511 | +408  +326, +490 | +477  +393, +562 | +479  +394, +565 | +459  +373, +545 |
| ^a^ Two referent group approaches were employed: referent month = month -3 and referent month = month -3 to month -1 mean.  ^b^ Difference in cost compared to referent; positive difference indicates an increase over referent.  Abbreviations: CI, confidence interval; Diff, difference; PPPM, per patient per month; USD, US dollars. | | | | | | | | | | | | | | | |
